# Cerebral Metastasis from a Previously Undiagnosed Appendiceal Adenocarcinoma

**DOI:** 10.1155/2012/192807

**Published:** 2012-11-01

**Authors:** Antonio Biroli, Paolo Cipriano Cecchi, Susanne Pragal, Esther Hanspeter, Andreas Schwarz

**Affiliations:** ^1^Department of Neurosurgery, University Hospital, 37126 Verona, Italy; ^2^Operative Unit of Neurosurgery, Regional General Hospital, Via L. Boehler 5, 39100 Bolzano, Italy; ^3^Internal Medicine, General Hospital, 39028 Silandro, Italy; ^4^Service of Pathology, Regional General Hospital, 39100 Bolzano, Italy

## Abstract

Brain metastases arise in 10%–40% of all cancer patients. Up to one third of the patients do not have previous cancer history. We report a case of a 67-years-old male patient who presented with confusion, tremor, and apraxia. A brain MRI revealed an isolated right temporal lobe lesion. A thorax-abdomen-pelvis CT scan showed no primary lesion. The patient underwent a craniotomy with gross-total resection. Histopathology revealed an intestinal-type adenocarcinoma. A colonoscopy found no primary lesion, but a PET-CT scan showed elevated FDG uptake in the appendiceal nodule. A right hemicolectomy was performed, and the specimen showed a moderately differentiated mucinous appendiceal adenocarcinoma. Whole brain radiotherapy was administrated. A subsequent thorax-abdomen CT scan revealed multiple lung and hepatic metastasis. Seven months later, the patient died of disease progression. In cases of undiagnosed primary lesions, patients present in better general condition, but overall survival does not change. Eventual identification of the primary tumor does not affect survival. PET/CT might be a helpful tool in detecting lesions of the appendiceal region. To the best of our knowledge, such a case was never reported in the literature, and an appendiceal malignancy should be suspected in patients with brain metastasis from an undiagnosed primary tumor.

## 1. Introduction

Brain metastases arise in 10%–40% of all cancer patients and are the commonest intracranial tumor [1, 2]. According to different series in the literature, up to one third of these patients do not have previous cancer history [2–4]. This latter group of patients includes those in whom primary tumor is detected during the initial diagnostic workup and also cases where the source of the metastasis, at least initially, remains undetected after systemic investigation. 

Primary appendiceal neoplasms are a rare group of malignancy that constitutes less than 0.5% of all tumors of gastrointestinal origin [5]. We report an extremely rare case, to the best of our knowledge never described in the literature, of a patient who presented with a right temporal lobe metastasis from a previously unknown appendiceal carcinoma.

## 2. Case Presentation

**Figure 1:**
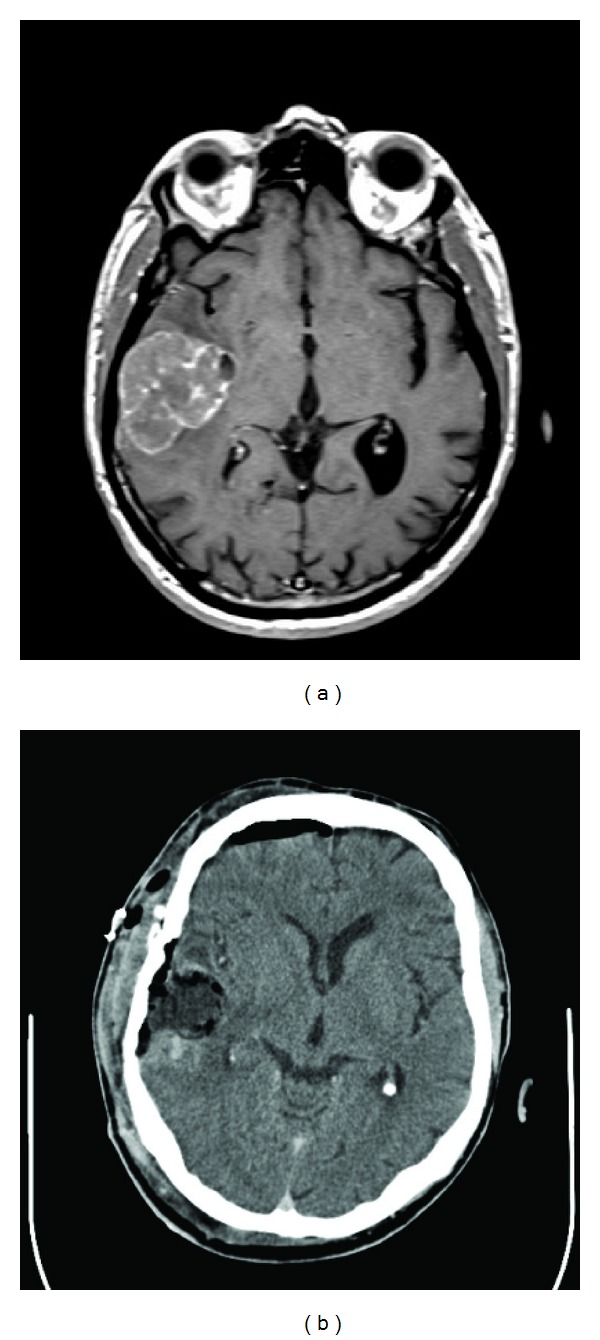
(a) Preoperative T1 axial contrast MR images showing a right temporal lesion with surrounded edema. (b) Postoperative head CT scan showing gross-total resection of the lesion.

**Figure 2:**
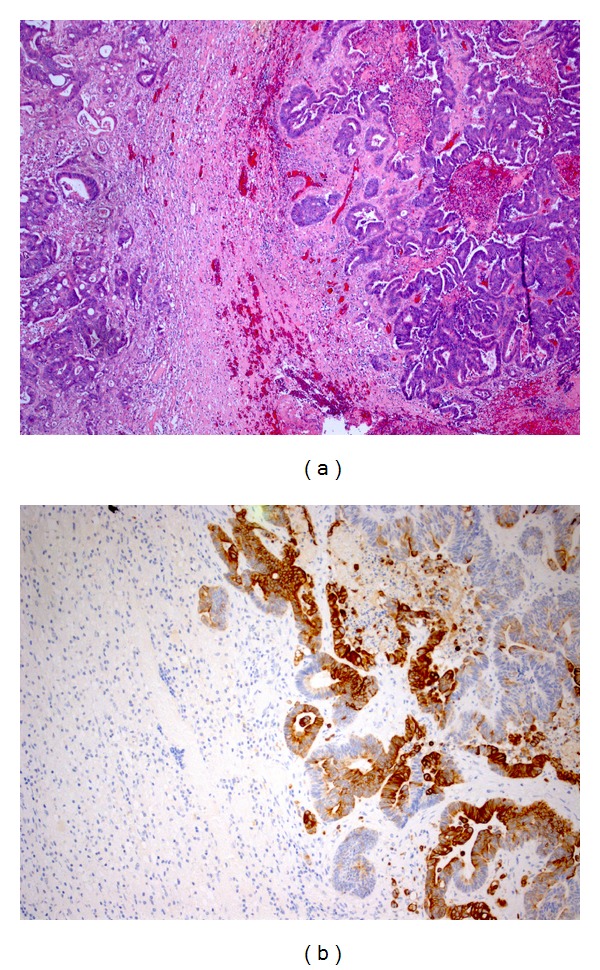
(a) Brain tissue infiltrated by an adenocarcinoma, original magnification ×40. (b) Immunohistochemical cytoplasmic stain for CK 20, a low-grade cytokeratin typical for colonic origin, original magnification ×100.

**Figure 3:**
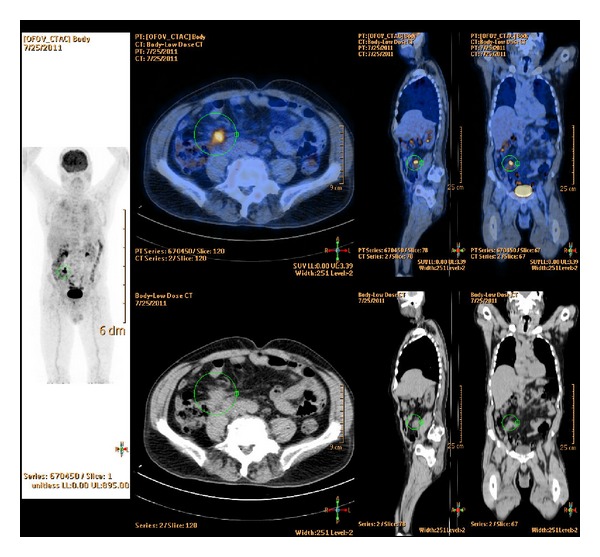
F-18 FDG PET/CT showing enhancement and intense abnormal tracer uptake focus in the appendiceal nodule in front of the right psoas major.

A 67-years-old male patient presented to our attention complaining of confusion, tremor and apraxia. His past medical history was unremarkable. On admission, neurological examination was normal. CT scan of the head and subsequent MRI of the brain revealed an isolated right temporal lobe lesion of 4 cm in its major axis, with inhomogeneous contrast enhancement and extensive surrounding edema (Figure 1(a)) both compatible with a brain metastasis or a high-grade glioma. A thorax-abdomen-pelvis CT scan showed no primary neoplastic lesion. Few days later, the patient underwent a right temporal craniotomy with gross-total resection of the lesion, as documented by postoperative head CT scan (Figure 1(b)). Postoperative course was uneventful. Histopathological examination revealed an adenocarcinoma of intestinal type with an immunohistochemical pattern compatible with a gastrointestinal origin (positive reaction to Cd-X2 and Ck-20 antigens and negative reaction to Napsin A and TTF19 antigens) (Figures 2(a) and 2(b)). A colonoscopy showed no evident primary lesion. A PET-CT scan showed elevated FDG uptake in the appendiceal nodule (Figure 3), and therefore an ileocecal resection with right hemicolectomy was performed. Histopathological specimen showed a moderately differentiated mucinous appendiceal adenocarcinoma with regional lymph nodes and peritoneal invasion (pT4a N1 M1 G2 R0 V0 L1). Whole brain radiotherapy (WBRT) using a conventional fractionation with a daily dose of 2 Gy and a total dose of 30 Gy was administered. A subsequent thorax-abdomen CT scan revealed multiple lung and hepatic metastasis, but the patient and his relatives refused any form of further oncological treatment. Seven months later, the patient died of disease progression.

## 3. Discussion 

Brain metastases arise in 10%–40% of all cancer patients and are a significant source of morbidity and mortality, with a median survival ranging from 7 to 12 months [1, 6]. Most common primary sites are lung (52.3%), followed by breast (8.9%), renal (5.4%), rectum (5.2%), gastric (5.2%), and colon (4.1%) [6]. According to different series, at initial presentation, up to 63% of the patients have multiple tumors, while 37%–50% present with a single brain metastasis [7, 8]. Brain metastases usually occur at the grey-white junction and in the watershed areas of brain at the same sites as cerebral emboli. The usual distribution is about 80% cerebral hemisphere, 15% cerebellum, and 3% brainstem, similar to blood flow [8]. 

To date, there are no standard guidelines regarding the various treatment options (WBRT, stereotactic radiosurgery and surgery differently combined) or data concerning overall survival and clinical outcome [1, 9, 10], especially for single brain metastatic lesions.

We described an extremely rare case (to the best of our knowledge never reported in the literature) of brain metastasis from a previously unknown appendiceal adenocarcinoma.

Presentation with a brain metastasis from a previously undiagnosed primary tumor is not a rare event [4]. This definition includes both patients in whom brain metastasis is the first lesion clinically symptomatic (as in our case) in association with a primary neoplasm previously unknown and patients whose primary tumor remained undetected even after a detailed systemic investigation. In the latter group, patients are usually in better general condition and the most common site of primary tumor is the lung, while gastrointestinal tract (GI) was involved in about 3%–10% of the cases [2–4]. In up to 14% of the patients primary tumor will remain undiagnosed [4], sometimes even at autopsy (3%) [3]. Some common types of primary metastatic tumor, such as breast carcinoma and melanoma, were significantly rare in this group of patients [3, 4]. Overall median survival ranged from 6 to 10 months. Eventual identification of the primary tumor does not affect survival, and delaying treatment intervention in pursuit of a primary diagnosis may not be appropriate [2–4, 11, 12]. 

Although brain metastases from all tumors of GI origin, generally a late event in the cancer history (only 11.1% had brain involvement as their initial presentation), occur in less than 4% of all cases, their incidence is increasing probably because of more effective systemic treatments and prolonged survival. Median survival ranges from 3.8 to 5.4 months. Young age (<65 years old), good Karnofsky performance status, presence of a solitary cerebral lesion, absence of systemic disease at presentation, and type of treatment (steroid, WBRT, and stereotactic radiosurgery and chemiotherapy) seemed as factors associated with prolonged survival [13, 14]. 

Primary adenocarcinoma of the appendix is a rare malignancy that constitutes less than 0.5% of all GI neoplasms and is generally difficult to diagnose preoperatively [5, 15, 16]. Appendiceal abnormalities are infrequently seen on colonoscopy and rarely yield a diagnostic biopsy in patients with appendiceal carcinoma [17]. F-18 FDG PET/CT might be a helpful tool [18], as in our case. Being somewhat of a different entity, some authors proposed a division into 2 groups corresponding to 4 histological subtypes: carcinoids and noncarcinoids appendiceal neoplasm (adenocarcinoma, mucinous adenocarcinoma, and adenosquamous carcinoma) [5]. Local spread to the peritoneum is a common event, whereas lymphatic or hematogenous metastases are only seen in 2% of appendiceal tumors [16]. Carcinoid tumors have little metastatic potential and very rarely present with metastasis. Noncarcinoid neoplasms have a more aggressive behavior. Overall 5-year survival was 83.1% for carcinoids versus 49.2% for noncarcinoid tumors. Beside histology and extent of the disease, tumor size was another prognostic factor [5, 15]. Mucinous adenocarcinoma (as in our case) has an incidence of 23%, is locally advanced in 90% of the cases, and is more likely to develop metastatic disease (usually peritoneal and ovarian) but has the highest survival rate among noncarcinoids group (51% 5 year survival) [5]. No standard treatment guideline has been established for primary appendiceal carcinoma due to its rarity and the heterogeneity of the disease right hemicolectomy seems superior compared to appendectomy in clinical outcomes but has no influence on patient survival, whereas only limited data are available on systemic chemotherapy for treating metastatic appendiceal carcinomas [5, 15, 19].

Our case report documents that appendiceal malignancy, even if very rare, should be suspected in those cases of cerebral metastasis with a previous unknown primary tumor.
